# Prognostic Effect of Subclassification on Oncological Outcomes in Patients with Surgically Treated Localized Papillary Renal Cell Carcinoma: A Retrospective Propensity Score-matched Cohort Study

**DOI:** 10.7150/jca.66916

**Published:** 2022-01-16

**Authors:** Shijie Li, Xuefeng Liu, Xiaonan Chen

**Affiliations:** Department of Urology, Shengjing Hospital of China Medical University, Shenyang, Liaoning 110004, People's Republic of China

**Keywords:** papillary renal cell carcinoma, clinical features, pathological features, prognosis, survival

## Abstract

**Background:** Few studies have reported on whether the histological subclassification of papillary renal cell carcinoma (PRCC) affects postoperative oncological outcomes. This study aimed to compare the clinical and pathological characteristics and outcomes of type 1 and type 2 PRCC patients undergoing surgical treatment at our hospital and to investigate the effect of PRCC histological subclassification on clinical outcomes.

**Methods:** We retrospectively analyzed the clinical and pathological data of 137 patients with PRCC who treated with surgery at our hospital between January 2007 and December 2020. Specifically, the clinical and pathological characteristics and oncological outcomes of 84 cases of type 1 PRCC and 53 cases of type 2 PRCC were analyzed. Propensity score matching was performed to minimize selection bias. The relationship between different subclassifications of PRCC and survival outcomes was applied by Kaplan-Meier survival analysis and multivariate Cox regression models.

**Results:** Median follow-up was 35 months. The 5-year overall survival (OS), cause-specific survival (CSS), and progression-free survival (PFS) of patients with type 1 PRCC were 95.5%, 97.0%, and 89.4%, respectively. The 5-year OS, CSS, and PFS of patients with type 2 PRCC were 78.6%, 83.3%, and 66.7%, respectively. The unmatched cohort showed that type 2 PRCC was associated with larger tumor diameters and more tumor thrombi. In the unmatched and matched cohorts, univariate analysis showed that smoking, pathological subclassification of type 2, pathological grade of G3/G4, and combination with tumor thrombus appeared to affect the outcomes of PRCC patients (*p* < 0.05). Multivariate analysis showed that smoking, pathological subclassification of type 2, and pathological grade of G3/G4 were independent risk factors for poor oncological outcomes with PRCC (*p* < 0.05). OS, CSS, and PFS were lower in type 2 PRCC than in type 1 PRCC in the unmatched and matched cohorts (*p* < 0.05). In addition, 3-year and 5-year OS nomograms were constructed based on the multivariate analysis, and the calibrated concordance index was high, indicating good calibration and feasibility for clinical practice.

**Conclusion:** Compared to type 1 PRCC, type 2 PRCC has significantly poorer OS, CSS, and PFS. History of smoking, histological subclassification, and pathological grade were independent predictors of oncological outcome. The nomogram based on histological subclassification was reliable for predicting the 3-year and 5-year OS of PRCC patients undergoing surgical treatment.

## Introduction

The worldwide incidence of renal cancer is increasing at an annual rate of 2.2%, and over 400,000 new cases are expected annually 1. Papillary renal cell carcinoma (PRCC) accounts for 15-20% of renal cell cancer cases and is a distinctly heterogeneous entity with different histological subclassifications, disease progression, and clinical outcomes 2. Traditionally, PRCC is subclassified into type 1 (pale cytoplasm, small cells) and type 2 (eosinophilic cytoplasm, large cells) PRCC 3. The 5-year overall survival (OS) rate of PRCC has been reported to be significantly higher than that of clear cell renal cell carcinoma (ccRCC) (80.5% vs. 71.3%) 4.

Early results have shown that type 2 PRCC has a higher nuclear grade, a more advanced stage, and a poorer prognosis compared with type 1 PRCC 5, 6. Recent research results show that type 1 and type 2 PRCC differ in their clinical and biological behaviors 7, 8. However, some researchers believe that histological subclassification has no effect on PRCC outcomes and that histological subclassification cannot be used as an independent prognostic determinant 9. Therefore, it remains unknown whether histological subclassification can be served as an independent prognostic factor; however, correct histological subclassification and outcome prediction are essential for medical planning. Meanwhile, nearly all these studies were conducted in Western populations, and these conflicting findings have not clearly revealed the clinical relevance of PRCC subclassifications in Asian populations.

This study aimed to analyze the long-term follow-up data of PRCC patients at a single center in China, to compare the differences in clinical and pathological characteristics between type 1 and type 2 PRCC patients undergoing surgical treatment, and to determine whether the histological subclassification of PRCC and other related factors can be used to predict postoperative outcomes.

## Patients and methods

### Patients

A total of 158 hospitalized patients diagnosed with PRCC at our hospital were continuously selected between January 2007 and December 2020. We included patients who (1) had type 1 or type 2 PRCC confirmed by pathology and (2) underwent radical nephrectomy or nephron-sparing surgery. The exclusion criteria were as follows: (1) incomplete clinical pathology and follow-up data (n = 12), (2) evidence of other malignant cancer during surgery (n = 2), and (3) absence of surgical treatment (n = 7). We selected patients in strict accordance with the predetermined criteria to ensure relative homogeneity of the selected patients. Finally, 137 eligible patients were included in the analyses. The clinical and pathological data and patient follow-up data were obtained from our institutional database. This descriptive study took place at Shengjing Hospital of China Medical University and was approved by the institutional review board. Written informed consent was obtained from all patients in the study.

### Study variables

To minimize potential selection bias, propensity score matching (PSM) was performed regarding key factors between the two groups. Patients were matched by the following variables: age, sex, body mass index (BMI), tumor laterality, smoking history, diabetes, hypertension, coronary heart disease, pT stage, pN stage, pM stage, tumor size, tumor grade, surgical approach, tumor thrombus, and hemoglobin. Enhanced CT of the urinary system was performed to determine the size and depth of tumor invasion. Cancer staging information was determined using the American Joint Committee on Cancer (AJCC) Staging Manual, 7th edition. Histological grade was determined by postoperative histopathological examination based on the Fuhrman nuclear grade. An experienced pathologist was assigned to re-examine the histologic subtypes of each PRCC according to the histopathologic criteria proposed by Delahunt and Eble 5. The histological characteristics of type 1 PRCC include a papillary surface covered with a single layer of cells, a small cell size, small nuclei, sparse cytoplasm, and basophilic staining. Type 2 PRCC includes a papillary surface covered with pseudostratified cells, with large cell volume, large nuclei, rich cytoplasm, and eosinophilic staining, with calcification and foamy macrophages in rare cases.

### Patient follow-up

All participants were followed up regularly through ongoing clinical assessments. In the first 2 years after surgery, follow-up examinations were generally performed every 3 months and included physical examination and laboratory examination. Tumor assessments were performed every 6 months using computed tomography (CT) and magnetic resonance imaging (MRI). If local recurrence or distant metastasis was suspected, imaging examinations including CT, MRI, bone scan, and positron emission tomography-CT (PET-CT) were performed immediately. OS was defined as time from study entry until death. Cancer-specific survival (CSS) was defined as the time interval between the date of surgery and death resultant from cancer. Progression-free survival (PFS) was defined as the time interval between the start of treatment and the first disease recurrence, progression, or death from any cause. Patient follow-up continued until death or December 2020.

### Statistical analysis

Categorical variables are presented as counts and percentages. For variables conforming to the normal distribution, continuous variables are expressed as mean and standard deviation (SD); for variables with a skewed distribution, continuous variables are expressed as median and interquartile range (IQR). The chi-square test, one-way analysis of variance, and Mann-Whitney U test were used to compare the association between categorical variables, normally distributed variables, and non-normally distributed variables, respectively. Cox regression analysis and PSM were used to minimize potential treatment allocation bias and confounding bias, and the 1:1 nearest-neighbor matching algorithm was used with a caliper width of 0.01. The receiver operating characteristic (ROC) curve and area under curve (AUC) were used to evaluate the reliability of the PSM scores. We conducted a series of sensitivity analyses to evaluate the robustness of the study results and how the application of various relational learning models would affect our conclusions. In the sensitivity analyses, we applied five relational learning models: the original uncorrected model, a multifactor calibration model, a PSM model, an inverse probability of treatment-weighting (IPTW) regression analysis model, and a standardized mortality ratio-weighting (SMRW) regression analysis model. The effect size and *p*-value of all models were calculated, reported, and compared. Multifactor adjustment, matching, weighting, and other methods were used to balance the diagnostics of the matching between the two groups. Kaplan-Meier curves (using the log-rank test) were performed to estimate the influence of clinical and pathological factors on OS, CSS, and PFS in the two groups before and after PSM. In the univariate results, *p* < 0.05 was used as the standard for inclusion into the multivariate Cox regression survival analysis to determine the independent association between the clinical characteristics and survival. In addition, a nomogram was constructed based on the results of multivariate Cox proportional hazards analysis to predict the 3- and 5-year OS. Discrimination was evaluated using the concordance index (C-index) and AUC. Calibration refers to how predictions from the nomogram compare to the observed outcomes. Decision curve analysis (DCA) was proposed to assess the net benefit of our model.

Analyses were performed using statistical software (R version 4.0.2; http://www.r-project.org/). Differences were considered statistically significant if two-sided P values were <0.05.

## Results

### Patient survival

**Figure 1:**
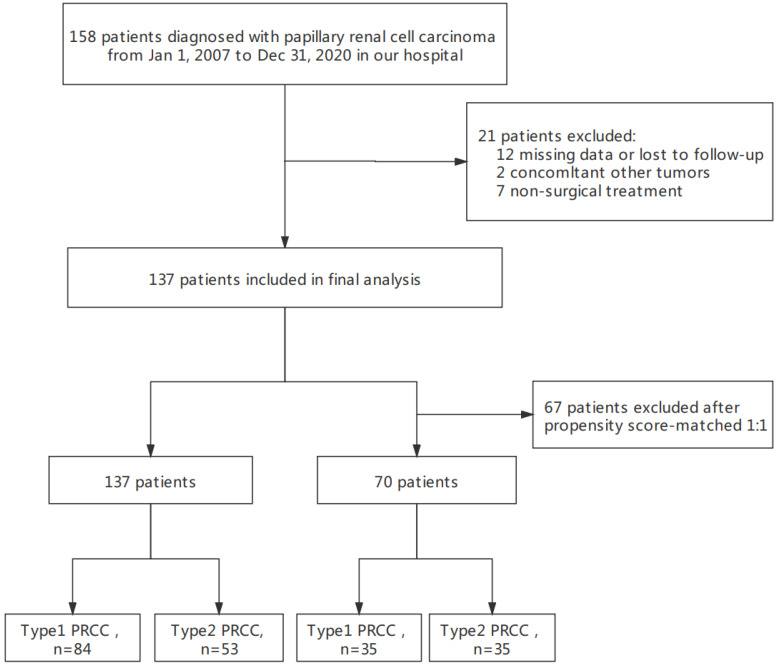
The flow chart of patient selection.

**Figure 2:**
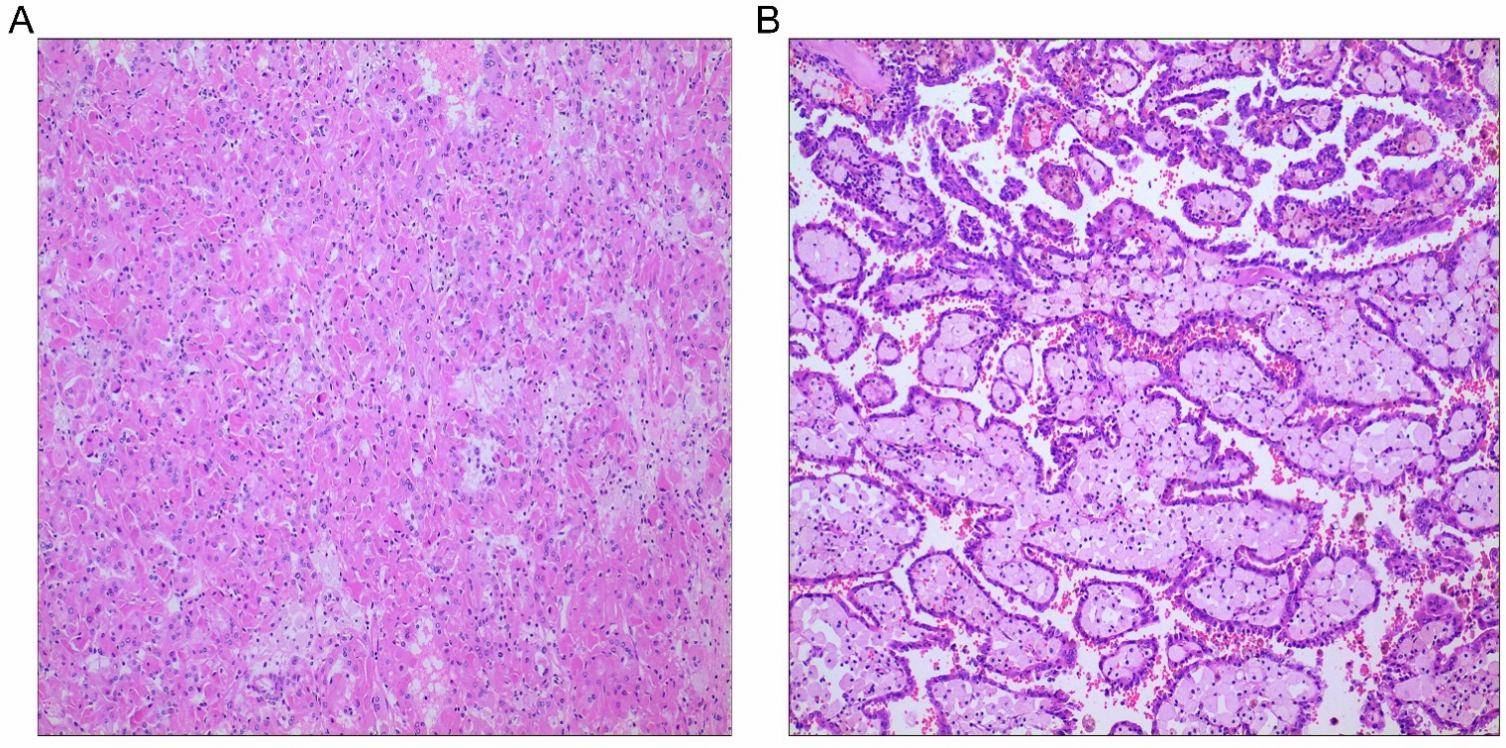
PRCC subtypes. A, PRCC type 1; B, PRCC type 2; Hematoxylin and eosin stains, original magnification ×100.

This final study cohort included 137 patients with PRCC who underwent nephron-sparing surgery or radical nephrectomy at our hospital. The flowchart of the study is shown in *Fig. 1.* Specifically, there were 84 cases of type 1 PRCC and 53 cases of type 2 PRCC based on the histopathologic criteria (*Fig. 2A and B*). Thirty-five pairs were obtained after PSM.

During a mean follow-up of 35 (IQR: 4-129) months, 9 of 137 patients (6.6%) died of UTUC. Postoperative tumor progression was confirmed in 24 patients (17.5%) and all-cause death was recorded in 12 patients (8.8%). For the entire cohort, the 5-year OS was 89.0%, CSS was 91.7%, and PFS was 80.7%. The 5-year CSS, OS and PFS of patients with type 1 PRCC were 97.0%, 95.5% and 89.4%, respectively. The 5-year CSS, OS and PFS of patients with type 2 PRCC were 83.3%, 78.6% and 66.7%, respectively. Kaplan-Meier analysis illustrated that the differences were statistically significant.

### Baseline clinicopathologic features between type 1 and type 2 PRCC

Table 1 shows the baseline characteristics of patients stratified by histological subclassification before and after PSM. Before PSM, there were significant differences in tumor diameter and tumor thrombus status between different histological subclassifications. The type 2 PRCC group tended to have a larger tumor diameter (*p* = 0.002) and more tumor thrombi (*p* = 0.014) compared to the type 1 PRCC group. No significant differences were found in age, sex, BMI, tumor laterality, smoking history, diabetes, hypertension, coronary heart disease, pT stage, pN stage, pM stage, tumor grade, surgical approach, and hemoglobin level.

**Figure 3:**
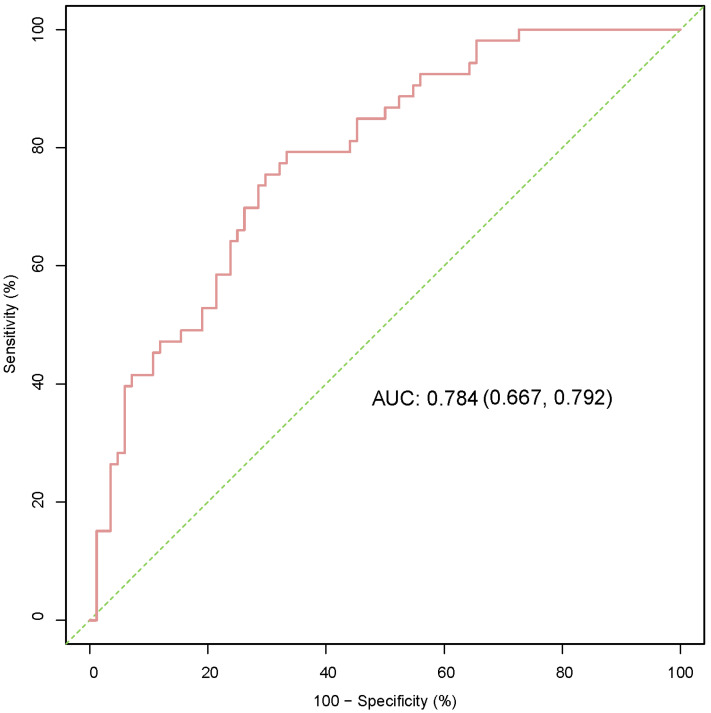
ROC-AUC analysis was used to evaluate the reliability of the PSM score. ROC, receiver operating characteristic; AUC, area under curve; PSM, propensity score-matched.

**Figure 4:**
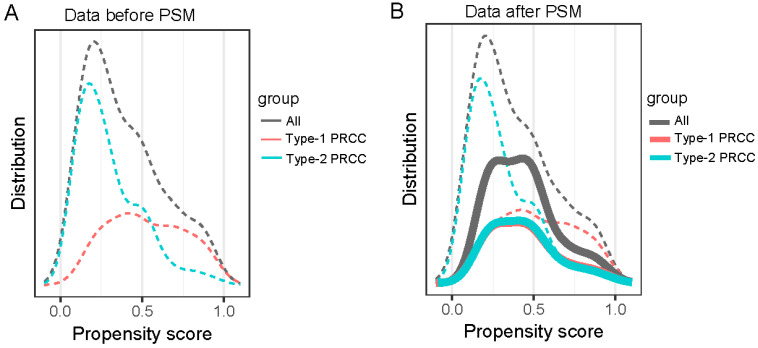
The covariate balance figure showed that the baseline characteristics of the different histological subclassification groups after PSM were well matched. (A)Data before PAM; (B)Data after PAM. PSM, propensity score-matched.

We formed a set of matched patients with similar propensity score values (1:1 match), excluding patients with no PSM. Our final study population consisted of 70 patients (35 per group). Among these patients, there were 52 men (74.3%) and 18 women (25.7%), with an average age of 55.7 ± 13.4 years. Fifteen patients (21.4%) had a clinical stage of T3/T4, and 7 patients (10.0%) had a pathological stage of G3/G4. After PSM, the baseline variables between the two groups were balanced. The clinical and pathological parameters did not differ significantly between the groups, suggesting balanced matching between the two groups. ROC-AUC analysis (*Fig. 3*) showed a PSM score of 0.784, indicating reliability of the matching method. The covariate balance figure showed that the baseline characteristics of the different histological subclassification groups after PSM were well matched (*Fig. 4A, B*). For sensitivity analysis, the same conclusion was reached in all five evaluation models (Table 2): the outcomes of type 2 PRCC were significantly worse than those of type 1 PRCC.

### Effect of histological type on survival outcomes

Before matching, univariate analysis (Table 3) showed that smoking history, pathological subclassification of type 1, pathological grade of G3/G4, and tumor with tumor thrombus appeared to affect the outcomes of patients with PRCC. Multivariate analysis (Table 4) showed that smoking history (HR = 6.43, 95% CI = 1.13-36.67, *p* = 0.036), pathological subclassification of type 2 (HR = 8.19, 95% CI = 1.55-43.18, *P* = 0.013), and pathological grade of G3/G4 (HR = 18.3, 95% CI = 3.15-106.24, *p* = 0.001) were independent risk factors for poor oncological outcomes.

**Figure 5:**
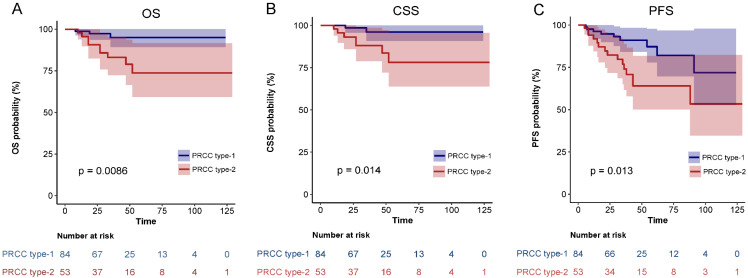
Comparison of Kaplan-Meier survival curves for OS(A), CSS(B), PFS(C) of localized type I and type II PRCC before PSM. CSS, cancer-specific survival; OS, overall survival; PFS, progression free survival; PRCC, papillary renal cell carcinoma; PSM, propensity score-matched.

**Figure 6:**
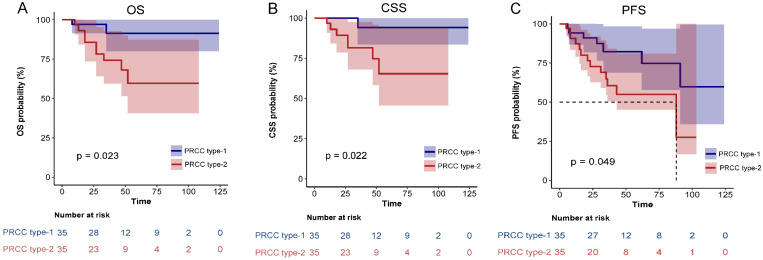
Comparison of Kaplan-Meier survival curves for OS(A), CSS(B), PFS(C) of localized type I and type II PRCC after PSM. CSS, cancer-specific survival; OS, overall survival; PFS, progression free survival; PRCC, papillary renal cell carcinoma; PSM, propensity score-matched.

After matching, univariate analysis showed that smoking history, diabetes, pathological subclassification of type 2, pathological grade of G3/G4, and tumor with tumor thrombus appeared to correlate with patient prognosis (*Table 3*). Multivariate analysis showed that smoking history (HR = 21.65, 95% CI = 1.89-248.19, *p* = 0.013), pathological subclassification of type 2 (HR = 10.24, 95% CI = 1.09-96.04, *p* = 0.042), and pathological grade of G3/G4 (HR = 30.6, 95% CI = 2.92-320.83, *p* = 0.004) were independent risk factors for poor oncological outcomes (*Table 4*). Kaplan-Meier survival analysis indicated that patients with type 2 PRCC had significantly lower OS, CSS, and PFS compared with those with type 1 PRCC (*p* < 0.05) in both the unmatched (*Fig. 5*) and matched cohorts (*Fig. 6*).

### Prognostic nomograms for OS

**Figure 7:**
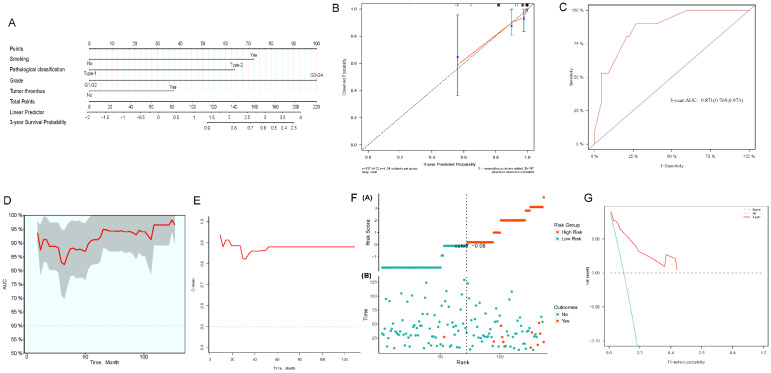
Nomogram constructed by independent prognostic factors predicting 3-year OS for surgically treated localized PRCC patients(A); corresponding calibration curves of nomogram for 3-year OS (B); the ROC curve of histological subclassification-based nomogram for predicting 3-year OS (C); time-dependent AUC of 3-year OS for the cohort (D); time-dependent C-index of 3-year OS for the cohort (E); the risk score graph reflected a high accuracy in assessing patient prognosis (F); DCA curves detect the clinical utility of the nomogram in patient prognosis (G). OS, overall survival; AUC, area under curve; DCA, decision curve analysis; PRCC, papillary renal cell carcinoma.

**Figure 8:**
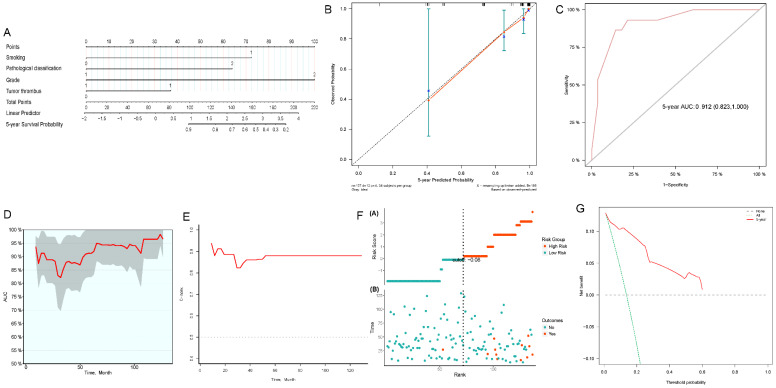
Nomogram constructed by independent prognostic factors predicting 5-year OS for surgically treated localized PRCC patients(A); corresponding calibration curves of nomogram for 5-year OS (B); the ROC curve of histological subclassification-based nomogram for predicting 5-year OS (C); time-dependent AUC of 5-year OS for the cohort (D); time-dependent C-index of 5-year OS for the cohort (E); the risk score graph reflected a high accuracy in assessing patient prognosis (F); DCA curves detect the clinical utility of the nomogram in patient prognosis (G). OS, overall survival; AUC, area under curve; DCA, decision curve analysis; PRCC, papillary renal cell carcinoma.

We constructed 3- and 5-year nomograms for OS based on histological classification (*Fig. 7A, 8A*) considering the results of the multivariate Cox regression analysis. The calibration plot, which runed very close to the diagonal, showed excellent calibration between the probabilities predicted by the nomogram and the actual observed results at 3 and 5 years after surgery (*Fig. 7B, 8B*). The AUC of the 3- and 5-year nomograms were 0.871 (95% CI: 0.769-0.973; *Fig. 7C*) and 0.912 (95% CI: 0.823-1.000; *Fig. 8C*), respectively. Time-dependent AUC (*Fig. 7D, 8D*) and C-index (*Fig. 7E, 8E*) results indicated that the nomogram had high accuracy. *Figure 7F and 8F* showed that the risk score graph reflected the high accuracy of our model in assessing the prognosis of patients. DCA revealed that the nomogram had a practical range of threshold probabilities and significant net benefit (*Fig. 7G, 8G*), and had clinical application for obtaining greater benefit and predicting individualized survival outcomes of PRCC patients.

## Discussion

In this retrospective, propensity score-matched cohort study, the relationship between PRCC of different histological subclassifications and survival outcomes was investigated. We found that type 2 PRCC tumors were larger in diameter and had a higher number of cases with tumor thrombi than type 1 PRCC. This indicated that the course of type 2 PRCC was often more aggressive than that of type 1 PRCC, consistent with the results of previous studies 10. With respect to prognostic factors, both univariate and multivariate analyses showed that in unmatched and matched patient cohorts, different histological subclassifications were independent predictors of CSS, OS, and PFS.

PRCC is the largest subgroup of non-clear cell renal carcinoma. Most previous papers have reported that PRCC usually has better outcomes than ccRCC 11, 12. However, in other studies, the outcomes of PRCC were comparable or even worse than those of ccRCC 13, 14. These conflicting results may be due to several reasons. First, because PRCC is rarer than ccRCC, the difference in the number of cases included in different studies may contribute to different results. Second, the widespread use of clinically targeted drugs such as sunitinib and sorafenib in recent years has significantly improved the prognosis of ccRCC. However, the efficacy of targeted drugs in PRCC is poorer than in ccRCC 15. Third, recent studies on the relationship between PRCC histological subclassifications and prognosis had relatively small sample sizes and a relatively insufficient follow-up period, which may also have largely impacted the results.

The oncological outcomes of kidney cancer are associated with certain clinical and pathological parameters. In the present study, we performed PSM to reduce the impact of characteristic differences between baseline clinical and pathological parameters in order to obtain convincing and statistically significant results. We found that smoking history, diabetes, pathological subclassification, pathological grade, and tumor with tumor thrombus appeared to correlate with patient prognosis. Multivariate analysis showed that smoking history, pathological subclassification of type 2, and pathological grade of G3/G4 were independent risk factors for poor oncological outcomes. The present study suggested that the OS, CSS, and PFS were lower in type 2 PRCC than in type 1 PRCC in non-matched and matched cohorts, considering that pathological subclassification can be used as an independent prognostic factor in kidney cancer. The results are consistent with those of the studies by Ren *et al.* 12 and Pignot *et al.* 16, suggesting that histological subclassification is an important prognostic factor in patients with PRCC. Considering the paucity of PRCC cases and the limitations of existing studies, the present study provides new evidence concerning this problem, which holds major research value. The identification and comprehensive early diagnosis of type 2 PRCC are crucial for the adjuvant treatment of locally advanced cancers or those with a poor prognosis.

In the present study, we also identified that tumor grade is an independent predictor of survival. The OS, CSS, and PFS of patients with high-grade PRCC were significantly reduced. Similar to the conclusions of the present study, a multicenter study by Zucchi *et al.* 17 found that Fuhrman nuclear grade was the primary independent predictor of outcomes associated with PRCC. Therefore, both pathological subclassification and pathological grade may have significant impacts on prognosis.

Recently, nomograms have been widely used for the clinical prediction of oncological outcomes of various cancers, such as breast, colorectal, stomach, and kidney cancer 18-21. There are few nomograms used for PRCC, and even fewer for cases of PRCC that have undergone surgical treatment. Therefore, we constructed a novel nomogram for PRCC patients undergoing surgical treatment using independent prognostic factors including histological subclassifications. The nomogram can be used to help predict the patient's individualized oncological outcomes and identify PRCC with aggressive clinical behavior, which may help individualize postoperative monitoring and treatment.

Although the present study provides new information for answering questions about the prognosis of patients with type 1 and type 2 PRCC, it still has potential limitations. First, because it was a retrospective study and not a randomized controlled trial, there may have been unobserved confounding because of the nature of retrospective studies, which might have affected the results. In the present study, we adjusted for probable confounding factors as much as possible and balanced them in a cohort with PSM. Second, this was a retrospective analysis from an observational cohort study, and our findings are therefore limited to associations. Our study was limited by a short overall follow-up period and a small sample size, which weakened the statistical power. Despite these limitations, understanding the results of the present study is vital to PRCC research. Third, many new markers associated with the clinical endpoints of PRCC have been identified in recent years 22-24; however, our nomograms did not include molecular markers. We believe that they should be included in predictive tools only when they add important information to existing factors and can change clinical decision-making. Until molecular data are validated and testing becomes common and cost-effective, it is still necessary to use only the latest nomograms comprising clinical and pathological characteristics for predicting the outcome of PRCC patients undergoing surgical treatment. Finally, the accuracy of the nomograms has not been externally verified and the main results and conclusions of the study in this paper still need further validation. A multicenter, prospective study on PRCC with more samples is still required.

## Conclusions

Type 2 PRCC was associated with a larger tumor diameter and more tumor thrombi compared with type 1 PRCC. OS, CSS, and PFS were lower in type 2 PRCC patients than in type 1 PRCC patients in the unmatched and matched cohorts. Smoking history, histological subclassification, and pathological grade were independent predictors of oncological outcomes. The histological subclassification-based nomogram had good predictive value for the prognosis of PRCC patients undergoing surgical treatment. However, prospective, large-sample, multicenter studies are still required.

## Figures and Tables

**Table 1:** Baseline characteristics of type-1 and type-2 patients with PRCC in the unmatched and matched groups.

Variables	Unmatched Patients				Propensity-Score-Matched Patients			
	Total (n = 137)	Type-1 (n = 84)	Type-2 (n = 53)	*P-*value	Total (n = 70)	Type-1 (n = 35)	Type-2 (n =35)	*P*-value
Age, Mean ± SD	56.3 ± 13.0	56.5 ± 12.8	56.0 ± 13.4	0.827	55.7 ± 13.4	55.1 ± 12.6	56.2 ± 14.4	0.731
Sex, n (%)				1				0.412
Male	101 (73.7)	62 (73.8)	39 (73.6)		52 (74.3)	28 (80)	24 (68.6)	
Female	36 (26.3)	22 (26.2)	14 (26.4)		18 (25.7)	7 (20)	11 (31.4)	
BMI, Mean ± SD	25.3 ± 5.3	24.7 ± 4.8	26.3 ± 5.9	0.102	25.8 ± 4.8	26.3 ± 5.5	25.2 ± 4.1	0.338
Laterality, n (%)				0.717				0.811
Left	71 (51.8)	42 (50)	29 (54.7)		36 (51.4)	19 (54.3)	17 (48.6)	
Right	66 (48.2)	42 (50)	24 (45.3)		34 (48.6)	16 (45.7)	18 (51.4)	
Smoking, n (%)				0.098				1
No	83 (60.6)	56 (66.7)	27 (50.9)		37 (52.9)	19 (54.3)	18 (51.4)	
Yes	54 (39.4)	28 (33.3)	26 (49.1)		33 (47.1)	16 (45.7)	17 (48.6)	
Diabetes, n (%)				1				1
No	129 (94.2)	79 (94)	50 (94.3)		67 (95.7)	34 (97.1)	33 (94.3)	
Yes	8 (5.8)	5 (6)	3 (5.7)		3 (4.3)	1 (2.9)	2 (5.7)	
Hypertension, n (%)				0.583				0.791
No	98 (71.5)	62 (73.8)	36 (67.9)		50 (71.4)	26 (74.3)	24 (68.6)	
Yes	39 (28.5)	22 (26.2)	17 (32.1)		20 (28.6)	9 (25.7)	11 (31.4)	
Coronary heart disease, n (%)				0.865				0.71
No	122 (89.1)	74 (88.1)	48 (90.6)		62 (88.6)	30 (85.7)	32 (91.4)	
Yes	15 (10.9)	10 (11.9)	5 (9.4)		8 (11.4)	5 (14.3)	3 (8.6)	
Tumor size (cm), Mean ± SD	4.9 ± 3.1	4.3 ± 2.6	5.9 ± 3.5	0.002*	5.2 ± 2.9	5.4 ± 3.1	5.1 ± 2.8	0.697
pT-stage, n (%)				0.138				1
T1/T2	113 (82.5)	73 (86.9)	40 (75.5)		55 (78.6)	28 (80)	27 (77.1)	
T3/T4	24 (17.5)	11 (13.1)	13 (24.5)		15 (21.4)	7 (20)	8 (22.9)	
pN-stage, n (%)				0.107				1
No	126 (92.0)	80 (95.2)	46 (86.8)		64 (91.4)	32 (91.4)	32 (91.4)	
Yes	11 (8.0)	4 (4.8)	7 (13.2)		6 (8.6)	3 (8.6)	3 (8.6)	
pM-stage, n (%)				1				1
No	132 (96.4)	81 (96.4)	51 (96.2)		70 (100.0)	35 (100)	35 (100)	
Yes	5 (3.6)	3 (3.6)	2 (3.8)		0 (0.0)	0 (0.0)	0 (0.0)	
Grade, n (%)				0.865				1
G1/G2	122 (89.1)	74 (88.1)	48 (90.6)		63 (90.0)	32 (91.4)	31 (88.6)	
G3/G4	15 (10.9)	10 (11.9)	5 (9.4)		7 (10.0)	3 (8.6)	4 (11.4)	
Surgery approach, n (%)				0.188				1
Open surgery	64 (46.7)	35 (41.7)	29 (54.7)		33 (47.1)	17 (48.6)	16 (45.7)	
Laparoscopic surgery	73 (53.3)	49 (58.3)	24 (45.3)		37 (52.9)	18 (51.4)	19 (54.3)	
Tumor thrombus, n (%)				0.014*				0.428
Absent	127 (92.7)	82 (97.6)	45 (84.9)		63 (90.0)	33 (94.3)	30 (85.7)	
Present	10 (7.3)	2 (2.4)	8 (15.1)		7 (10.0)	2 (5.7)	5 (14.3)	
Hb, Median (IQR)	137.0 (118.0, 149.0)	138.0 (118.0, 148.2)	133.4 (119.9, 152.0)	0.951	138.0 (118.5, 150.5)	139.0 (130.0, 151.0)	130.0 (116.0, 148.0)	0.254

*Indicates *P*<0.05. PRCC, papillary renal cell carcinoma; BMI, body Mass Index; Hb, Hemoglobin.

**Table 2:** Evaluation of the robustness of the research results by five relational learning models: (1) original uncorrected model; (2) multifactor calibration model; (3) propensity score matching (PSM) model; (4) inverse probability of treatment-weighting (IPTW) regression analysis model (5) a standardized mortality ratio-weighting (SMRW) regression analysis model.

Item	HR (95%CI)	*P* (Wald's test)
Unmatched crude	4.88 (1.32,18.05)	0.017*
Multivariable adjusted	19.76 (2.09,187.32)	0.009*
Propensity Score Matched	5.01 (1.08,23.21)	0.04*
Weighted IPTW	3.8 (1.06,13.59)	0.04*
Weighted SMRW	4.15 (1.37,12.56)	0.012*

*Indicates *P*<0.05. PSM, propensity score-matched; IPTW; inverse probability of treatment-weighting; SMRW, standardized mortality ratio-weighting.

**Table 3:** Association between clinicopathological factors and OS in univariate analysis before and after PSM

Characteristics	Before PSM			After PSM	
	HR (95%CI)	*P*-value		HR (95%CI)	*P*-value
Age, years (Continuous)	0.99 (0.95,1.03)		0.554	1.0025 (0.9596,1.0473)	0.911
Sex (Female vs. male)	1.34 (0.4,4.46)		0.631	1.99 (0.58,6.84)	0.273
BMI, kg/m^2^ (Continuous)	0.99 (0.88,1.1)		0.801	0.97 (0.84,1.12)	0.694
Laterality (Right vs. Left)	1.08 (0.35,3.36)		0.888	1.3 (0.4,4.25)	0.668
Smoking (Yes vs. No)	5.28 (1.43,19.54)		0.013*	5.42 (1.17,25.11)	0.031*
Diabetes (Yes vs. No)	3.57 (0.78,16.34)		0.101	5.1 (1.1,23.68)	0.037*
Hypertension (Yes vs. No)	1.96 (0.62,6.19)		0.252	2.2 (0.67,7.22)	0.193
Coronary heart disease (Yes vs. No)	0.72 (0.09,5.62)		0.758	0.66 (0.08,5.19)	0.694
Pathological classification: Type 2 vs 1	4.88 (1.32,18.05)		0.017*	5.01 (1.08,23.21)	0.040*
Tumor size, cm (Continuous)	1.01 (0.86,1.19)		0.869	1.02 (0.84,1.22)	0.869
pT (T3/T4 vs. T1/T2)	1.3 (0.35,4.8)		0.696	1.09 (0.29,4.1)	0.903
pN (Yes vs. No)	0 (0, Inf)		0.998	0 (0, Inf)	0.998
pM (Yes vs. No)	0 (0, Inf)		0.998	-	-
Grade (G3/G4 vs. G1/G2)	4.71 (1.41,15.7)		0.012*	4.18 (1.1,15.91)	0.036*
Surgery (Open vs. Laparoscopic)	1.32 (0.42,4.18)		0.637	1.8 (0.52,6.22)	0.351
Tumor thrombus (Yes vs. No)	6.19 (1.85,20.74)		0.003*	5.01 (1.46,17.22)	0.010*
Hb, g/L (Continuous)	1.01 (0.98,1.03)		0.576	1.01 (0.98,1.05)	0.343
					

*Indicates *P*<0.05. OS, overall survival; BMI, body Mass Index; Hb, Hemoglobin; PSM, Propensity Score Matched.

**Table 4:** Association between clinicopathological factors and OS in multivariate analysis before and after PSM

Characteristics	Before PSM		After PSM	
	HR (95%CI)	*P*-value	HR (95%CI)	*P*-value
Smoking (Yes vs. No)	6.43 (1.13~36.67)	0.036*	21.65 (1.89~248.19)	0.013*
Diabetes (Yes vs. No)	-	-	1 (0.13~7.48)	0.999
Pathological classification: Type 2 vs 1	8.19 (1.55~43.18)	0.013*	10.24 (1.09~96.04)	0.042*
Grade (G3/G4 vs. G1/G2)	18.3 (3.15~106.24)	0.001*	30.6 (2.92~320.83)	0.004*
Tumor thrombus (Yes vs. No)	2.93 (0.75~11.36)	0.121	1.96 (0.37~10.4)	0.427

*Indicates *P*<0.05. OS, overall survival; BMI, body Mass Index; Hb, Hemoglobin; PSM, Propensity Score Matched.
